# Group Nutrition Counseling or Individualized Prescription for Women With Obesity? A Clinical Trial

**DOI:** 10.3389/fpubh.2020.00127

**Published:** 2020-04-30

**Authors:** Marciele Alves Bolognese, Carina Bertoldi Franco, Ariana Ferrari, Rose Mari Bennemann, Solange Munhoz Arroyo Lopes, Sônia Maria Marques Gomes Bertolini, Nelson Nardo Júnior, Braulio Henrique Magnani Branco

**Affiliations:** ^1^Post-Graduation Program in Health Promotion, University Center of Maringa, Maringa, Brazil; ^2^Research Group in Physical Education, Physiotherapy, Sports, Nutrition and Performance of the University Center of Maringa (GEFFEND/UniCesumar), Maringa, Brazil; ^3^Medicine Department, University Center of Maringa, Maringa, Brazil; ^4^Post-Graduation Program in Science, Technology and Food Safety, University Center of Maringa, Maringa, Brazil; ^5^Department of Physical Education, State University of Maringa, Maringa, Brazil

**Keywords:** multiprofessional research, health promotion, mental health, obesity, women's health

## Abstract

**Background:** This study investigates the effects of group counseling vs. individual dietary prescription on physical, nutritional, and mental health in overweight or obese women.

**Methods:** Seventy-four women aged 40–59 years with body mass index ≥ 25 kg/m^2^ were randomized into 2 intervention arms: group nutrition counseling (GNC) or individualized nutrition prescription (INP). Twenty-seven women completed the 12-week intervention protocol. The GNC received counseling once a week and the INP received an individualized prescription once a month. All participants attended physical exercise sessions 3 times a week following the same protocol. Body mass, body mass index, fat mass, body fat percentage, lean mass, lipid profile, hemoglobin A1c, insulin and liver transaminases were measured pre- and post-intervention in both arms. A 3 day food record was applied to calculate the intake of calories, carbohydrates, proteins, and lipids. Body image dissatisfaction, level of anxiety, self-esteem measure and pathological eating attitudes were measured.

**Results:** Both dietary interventions decreased body mass, body mass index, fat mass, body fat percentage, total caloric intake, carbohydrates, proteins, lipids, body dissatisfaction, anxiety, and saturated and polyunsaturated fats (*p* < 0.05). Lean mass, metabolic variables, self-esteem and pathological eating attitudes remained unchanged (*p* > 0.05).

**Conclusion:** Both nutritional interventions combined with concurrent exercise were effective to improve anthropometrics, body composition, food intake, and some mental health parameters. We suggest that the choice of nutritional intervention (GNC or INP) could be based on the participants preference, considering the adherence and satisfaction, to promote health and quality of life.

## Introduction

In Brazil, more than 50% of adults is overweight or obese (1). Obesity is a chronic, complex and multifactorial disease that is associated with increased risk of comorbidities such as type 2 diabetes (2), dyslipidemia (3) and cardiovascular disease (CVD) (4). In addition to affecting physical health, obesity has been associated with decrease in mental and social health (5). People with obesity have higher levels of anxiety, depression, binge eating, low self-esteem, social discrimination, disability with early retirement, and death (6, 7). Increasing prevalence of this condition is placing a major burden on the public health system with a large impact on the economy (4).

Several strategies have been used to minimize the impacts caused by obesity. Among these, lifestyle interventions that combine changes in eating behavior and regular exercise should be incorporated as a first step by those seeking weight loss (8–14). Current guidelines advocate the need for lifestyle changes, but calorie-restricted diets and/or macronutrient-manipulated diets continue to be the most widely used method to promote weight reduction and prevent CVD (9, 15).

However, it is noteworthy that the consumption of healthy food for a large portion of the Brazilian population is relatively expensive (16). Low-income families are more likely to choose unhealthy foods, which are industrialized and have high energy density contributing to increases in body mass index (BMI) and waist circumference, as well as a low self-assessment of health conditions (17). Therefore, cost-effective strategies for health promotion in overweight and obese people are essential and need to be incorporated by the Brazilian public health system (18, 19).

Although previous studies have shown that low-calorie diets and/or macronutrient manipulation are effective to promote short-term weight loss, it is not known whether one diet is more effective than others. Regarding weight loss and maintenance of lost weight, ~90–95% of treated people regain body mass over the time (20). On the other hand, Alvarenga et al. (21) and Wolever et al. (22) found that new nutritional strategies based on behavioral treatment without prescribing restrictive diets have satisfactory long-term results. For these authors, food does not perform only the function of nurturing physiological needs, but it goes beyond and fulfills the function of psychological and social needs. Thus, other perspectives for obesity treatment have emerged and were tested by the scientific community (18, 19).

It is noteworthy that overweight people, especially women, are more likely to have self-criticism and negative body image (23), which is stimulated by the standards of beauty imposed by society. The search for the ideal body promotes body image dissatisfaction and lower self-esteem, resulting in impairments in physical and mental health. Despite the constant efforts of those seeking weight loss, failure rates are high, which demonstrates how difficult it is to combat this twenty first century disease.

Another issue of weight loss intervention program is the adherence to diet changes. The literature points out that adherence rates can be very variable depending on the type of diet adopted and the individual wish for a lifestyle change (24). People often procrastinate the nutritional planning during a weight loss program, particularly women with poor health status (25). It is possible that, regardless of the nutritional counseling model, a close follow-up with the dietitian and health team can be the most effective factor to promote weight loss in people with obesity. People need time to change their eating habits and require continuous reinforcement to maintain a new lifestyle (24).

Given the worrisome prevalence of obesity, the number of strategies for weight loss and the high rates of failure, the aim of the present study was to investigate the effects of two different nutritional strategies: group nutrition counseling (GNC) or individualized nutrition prescription (INP), with a concurrent exercise program on the physical, nutritional and mental health of overweight or obese women after 12-weeks of intervention. As a hypothesis, it is believed that both nutritional strategies along with concurrent exercise would be effective to improve the health condition of overweight or obese women.

## Materials and Methods

### Participants

Women aged 40–59 years, with a BMI ≥ 25 kg/m^2^, and low-income (evaluated through a socioeconomic questionnaire) were included. All participants received medical clearance before starting physical exercises. The screening was based on the answers from the original sociodemographic questionnaire (more information is described below). We have decided to only include women from low-income backgrounds to reflect a major public health problem in our country. Brazil is a developing country with 54.8 million people living below the so-called income line ($5,5 a day) (26). As it is observed in other developing countries, excessive BMI is strongly related to poverty, particularly because of the high intake of ultra-processed foods (27, 28). The exclusion criteria were: (a) physical conditions that could limit the practice of physical activity; (b) diseases or history of use of drugs that affect body mass, body composition, or muscle strength; (c) postmenopausal hormone replacement therapy; (d) current smoking; (e) current participation in a program to reduce body mass or any type of diet; (f) <75% adherence to the interventions; and (g) failure to complete post-intervention assessments. This research used an experimental and longitudinal study of parallel groups and repeated measurements. Participants were recruited via non-probabilistic sampling, through advertisements on the television, radio, newspapers and internet, and in local basic health units. The sample size calculation identified that 9 participants per experimental group would be enough to detect differences in the dependent variables, with a smaller standard deviation when compared to previous studies with α = 0.05 and β = 80%. All participants were informed about the purposes of the present study and signed the informed consent form. The study was approved by the Ethics and Research Committee of the State University of Maringa by Opinion N^o^. 2,655,268/2018. This study was also submitted and approved by Brazilian Trials Clinical Studies (REBEC) by the Health Minister under register RBR-2YZS76.

### Study Protocol

**Figure 1:**
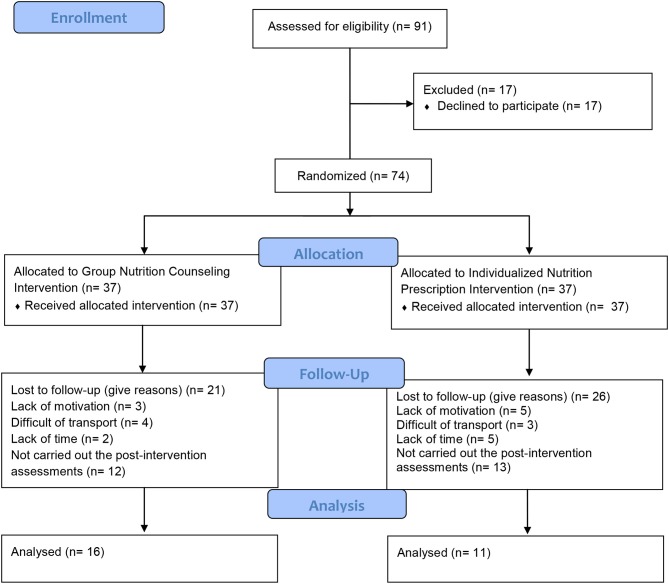
Flowchart of present study.

This study is a clinical trial with parallel groups and repeated measures. The participants were randomized via Excel software (version 2013, Microsoft, US), i.e., the participants were numbered and randomized and allocated to two different intervention groups: group nutrition counseling (GNC) or individualized nutrition prescription (INP), which are detailed below. During the 12-week intervention period, both groups attended sessions led by the nutrition team and received the same schedule of structured exercise training supervised by the kinesiology team, at the university facilities. The intervention protocol was developed by our team. First, medical clearance was performed, blood samples were collected for the measurement of metabolic and other variables (described below), and anthropometrical and body composition variables were assessed. All participants were given instructions to correctly fill out the 3 day food record, which was to be completed on 2 days during the week and 1 day on each weekend. Twenty-four hours later, the different questionnaires were completed (the details are described in the sections below). Thus, the different assessments were performed in 2 days, with 24 h of rest between them. The post-evaluation assessments were performed 2 days after the end of the intervention. In both protocols, the participants were attended by a certified nutritionist. The participants randomized to GNC received group counseling, whereas those in the INP received an individual prescription. Figure 1 presents the flowchart of the study.

### Sociodemographic Characteristics of the Participants

Sociodemographic characteristics were reported by participants via a questionnaire developed by the Brazilian Economic Classification Criteria (29). The following information was collected: age, education (classified by years of study as follows: illiterate, 1–4 years, 5–8 years, 9–11 years and 12 or more years of schooling), and economic class (considered classes A, B, C, and D). Economic class “A” represents higher family income, while economic class “D” represents lower family income (29).

### Anthropometric Assessment and Body Composition

After blood collection, all participants underwent anthropometric assessment with measurement of body mass and stature for the calculation of the BMI, as well as the assessment of body composition through a bioelectrical impedance. The evaluations took place 2 days prior to the exercise and nutritional intervention. The reevaluations followed the same protocol and were performed 2 days after the end of the 12-week intervention. Body mass (kg) and body composition were measured by InBody bioimpedance (model 570®, Body Composition Analyzers, Seoul, South Korea). Stature was measured using a Sanny® wall-mounted stadiometer (São Paulo, Brazil), capable of measuring up to 2.20 meters and accurate to within 0.1 cm. BMI was calculated as BMI = body mass (kg)/stature (m)^2^ and classified according to the WHO cut-off points (30). The participants were advised to follow these instructions before the assessment: (a) to abstain from alcohol for 12 h before the test, (b) to not perform high-intensity exercise for 12 h before the test, (c) to urinate 30 min before the test, (d) to fast at least 4 h before the assessment time, and (e) to wear light clothing free of metallic objects such as zippers; these instructions followed the recommendations by Heyward (31) and Branco et al. (32). The components of body composition used were the lean mass (LM), fat mass (FM), and body fat percentage (BF).

### Collection of Blood Samples to Measure Metabolic Tests

The blood samples were collected in a private laboratory with international ISO 9001 certification by a biomedical team without access to information about the nutritional intervention, and physical activity models. For the evaluation of metabolic parameters, 10 mL of median cubital vein blood was collected 12 h after fasting, with maintenance of the usual diet for 5 days before collection, no alcohol intake 72 h before collection, and no vigorous exercise within the previous 24 h. The following measures were assessed before and after the intervention period: (1) insulin; (2) glycated hemoglobin A1c (A1C); (3) triglycerides; (4) total cholesterol; (5) low-density lipoprotein (LDL-c); (6) high-density lipoprotein (HDL-c); and (7) liver transaminases (AST and ALT). For the diagnosis of dyslipidemia, the following criteria for at least one of the four analyzed variables needed to be met: total cholesterol ≥ 190 mg/dL, HDL-c ≤ 40 mg/dL, LDL-c ≥ 130 mg/dL, and triglycerides ≥ 150 mg/dL. The cut-off points used were those recommended by the American College of Cardiology Foundation (33). The references for A1C levels were <5.7% (normal), ≥ 5.7% and <6.5% (prediabetes), and ≥ 6.5% (probable diabetes) (2).

### Description of the GNC Protocol

The participants in GNC received nutrition information provided by the same registered nutritionist, once a week for an average of 40 min. They were given educational material about the contents presented and strategies to adapt their eating behaviors to different daily situations. All participants attended a meeting once a week during the intervention period (a total of 12 meetings). At the meetings, the following topics were addressed: (a) the intervention schedule; (b) the importance of maintaining a healthy body mass (use of behavioral nutrition); (c) the hunger odometer; (d) the food groups and their respective functions; (e) skills for challenging situations (use of nutritional and wellness coaching strategies); (f) reading food labels; (g) knowing the amount of sugar and fat in foods; (h) diet vs. light foods; (i) grocery shopping and meal planning; (j) mindfulness eating (cognitive behavioral therapy); and (k) how to transition to a new diet. In summary, the interventions were based on nutritional counseling and focused on changing eating behaviors adapted by Branco et al. (18, 19).

### Description of the INP Protocol

The participants in INP were followed monthly by a registered nutritionist, with consultations of ~1 h duration. The same nutritionist prescribed all meal planning for the participants in the INP group. The participants had an initial evaluation and fortnightly visits to adjust their eating plans, if necessary. The diet plan prescription was calculated based on the participants resting metabolic rate (RMR) obtained by InBody 570®, multiplied by 1.4, according to the Institute of Medicine (IOM) recommendations (34), for women with low levels of physical activity. The meal plan was individually delivered and comprised a basic menu including information such as mealtimes, food groups, and portions distributed between meals, along with a list of replacement groups and equivalent foods. It is noteworthy that the diet was not controlled in any of the experimental groups; that is, the information was provided to the participants, but adherence to the diet plan or the process of dietary re-education was not monitored on a daily basis. Importantly, the intervention groups did not have contact with each other including during the physical training sessions, which occurred on a different schedule.

### Food Record

The participants had previously been instructed by a nutritionist about how to complete the alternating 3 day food register: 2 days on weekdays and 1 day on the weekend (e.g., Tuesday, Thursday, and Saturday), according to the IOM (34). The participants were instructed to record all foods and beverages consumed, as well as their respective quantities and/or home measures, mealtimes, and places. They were also instructed to take notes right after each meal to avoid forgetting any of the information to be recorded (18, 19). Macronutrients, saturated fat, monounsaturated fat, polyunsaturated fat, total energy intake, and dietary fiber were calculated using the Avanutri nutritional calculation software program (version 2.0, Avanutri Assessment Equipment Ltd., Três Rios, Rio de Janeiro, Brazil). The Dietary Reference Intakes (DRIs) was used to assess nutrient intake adequacy of the participants (34).

### Questionnaires Applied to Assess the Level of Physical Activity and Mental Health

The following items were evaluated before and after 12-week intervention period:

- The IPAQ questionnaire validated for Brazilians was used to identify the levels of physical activity before and after the multiprofessional intervention, and to determine whether the women had already engaged in other moderate/intense physical activities before starting the program (35).
- Body image dissatisfaction was assessed by applying the body shape questionnaire (BSQ) (36), validated for the Brazilian population (37, 38). The BSQ is a questionnaire composed of 34 questions about body image dissatisfaction and concern with body measurements. Each question was given a value on a scale from 1 (never) to 6 (always). Higher levels of dissatisfaction were given higher scores. Based on the score, individuals are classified as satisfied (81 to 110 points) or dissatisfied (above 111 points) with body image.
- Anxiety level were assessed using the Hamilton Anxiety Scale (39), which aims to assess the severity of anxiety symptoms. The instrument consisted of 14 symptom groups, subdivided into two groups, seven related to anxious mood symptoms and seven related to physical anxiety symptoms. Responses ranged from zero to four, where 0 indicates the absence of a symptom, 1 indicates low average intensity, 2 indicates high average intensity, 3 indicates strong intensity, and 4 indicates disabling intensity. Scores ≥ 18 were defined as mild anxiety; ≥ 25, moderate anxiety; and ≥ 30, severe anxiety (40).
- Self-esteem was assessed with the Rosenberg self-esteem scale (RAS), which consisted of 10 statements related to a set of feelings of self-acceptance and self-esteem and assessed total self-esteem. The items are expressed in a four-point Likert scale ranging from strongly agree (3), agree (2), disagree (1) and strongly disagree (0). The alternatives are divided into five positive questions (1, 2, 4, 6, 7) and five negative questions (3, 5, 8–10). Each alternative has a value ranging from zero to three points. The higher the score, the higher the self-esteem of the individual. The final scale score can range from zero (low self-esteem) to thirty (high self-esteem) (41).
- Pathological eating attitudes screening was performed using the Eating Attitudes Test (EAT-26) (42), a questionnaire with 26 self-completion questions. The answers were evaluated by their score, giving three points for each item that was marked the most extreme answer (always), two points for the second most extreme answer (very often) and one point for the third most extreme answer (often), with the other answers given no points. Question number 4 has a particularity because it was reverse scored, i.e., “sometimes” was given 1 point, “rarely” was given 2 points and “never” was given 3 points, the other answers received no points; this occurred only for question 4. After application of the instrument, the scores obtained in each of the EAT-26 questions were summed for each person evaluated. A total score higher than 21 (twenty-one) confirmed the presence of pathological eating attitudes and the risk of an eating disorder (43).

### Description of Resistance-Training Protocol

Physical exercises were performed 3 times a week for ~47 to 62 min. Concurrent training was used, alternating resistance and aerobic exercises. In addition, the physical exercises followed the principles of pulling, pushing, knee dominance, and hip dominance, in addition to core work. First, physical exercises for large muscle groups were emphasized, and in the background, resistance exercises were performed for smaller muscle groups. The series alternated back and forth. The emphasis was given to work on muscle strength and endurance, flexibility, and cardiorespiratory fitness. Resistance exercises were accomplished using the participants' own body mass and the use of accessories such as TRX, medicine balls, rubber bands, Swiss balls, cones, an agility ladder, naval rope, tires, and steps. The use of these accessories is a low-cost method. The intensity was controlled using Borg scale 6–20 (44). The eccentric and concentric phases were stabilized at 1:1. The volume and intensity were measured during all exercise sessions. The physical trainers were blinded as to the types of nutritional care performed. Table 1 shows the physical exercise periodization during the 12-week intervention period.

**Table 1:** Physical exercises periodization during 12-week of intervention.

**Intensity**	**Sets**	**Time**	**Effort: pause ratio**	**Weeks**
10–12 a.u.	2 sets	52 min	40” per 20”	1st and 2nd
10–12 a.u.	2 sets	47 min	40” per 10”	3rd and 4th
12–14 a.u.	2 sets	52 min	50” per 10”	5th and 6th
12–14 a.u.	2 sets	62 min	60” per 20”	7th and 8th
15–17 a.u.	2 sets	60 min	60” per 15”	9th and 10th
15–17 a.u.	2 sets	58 min	60 per 10”	11th and 12th

*a.u., arbitrary units; effort, relation of time in seconds of physical effort; pause, time in seconds of rest*.

Table 2 shows the exercises performed during the 12-week training periodization.

**Table 2:** Training program performed during 12-week training periodization.

**Order**	**Training program A**	**Sets**
1	Warm-up - continuous and interval running	10 min
2	Plank with body weight	2x
3	Hip bridge	2x
4	Unipodal deadlifting with dumbbells	2x
5	Pulling tire with rope	2x
6	Standing calf raise	2x
7	Rope tsunami	2x
8	Mountain climber	2x
9	Lying twist	2x
10	Running at moderate-intensity and alternating with low intensity	5 min
11	General stretching	5 min
**Order**	**Training program B**	**Sets**
1	Warm-up—continuous and interval running	10 min
2	Plank with body weight	2x
3	Hip bridge	2x
4	In pairs: squat, lift and push the tire	2x
5	Squat—body weight	2x
6	Push-ups (on knees)	2x
7	Squat in isometric position	2x
8	Thruster with medicine ball	2x
9	Crunch abdomen	2x
10	Running at moderate-intensity and alternating with low intensity	5 min
11	General stretching	5 min

### Statistical Analysis

Initially, all data were tabulated in Excel software (version 2013, Microsoft, US). After data tabulation, statistical analyses were performed via Statistica (version 12.0, Stasoft, US). Data normality was tested using the Shapiro–Wilk test. After this confirmation, a two-way analysis of variance (ANOVA), group × time, was used, applying the Bonferroni test, if necessary. The significance level was established at 5% for all analyses. For ANOVA, Mauchly's sphericity test was employed, and the Greenhouse-Geisser correction was used, if necessary. For data presentation, the mean (±) standard deviation and the relative frequency of data from the food register were used. Finally, based on Cohen (45), the effect size was calculated according to the classification: up to 0.20 (*small effect*), from 0.20 to 0.80 (*medium effect*), and above 0.80 (*large effect*).

## Results

### General Characteristics

Table 3 shows that the mean age of the participants was 45.7 years old, and the level of education was heterogeneous, with a predominance of education equal to or lower than high school. In addition, the reported family income was mostly below 6 minimum salaries (96.28%).

**Table 3:** General characteristics and socioeconomic status of the participants of the present study.

**Variables**	**All: pre-intervention (*n =* 27)**	**GNC (*n=* 16)**	**INP (*n=* 11)**
Age (years old)	45.7 ± 3.2	45.9 ± 3.1	45.5 ± 3.3
**Scholarity level**			
Up to 9th grade	2 (7.40%)	1 (9.09%)	1 (6.25%)
High school	18 (66.67%)	8 (72.72%)	10 (62.5%)
University education	7 (25.92%)	2 (18.18%)	5 (31.25%)
**Socioeconomic level (ABEP)**			
A)> 6 salaries	1 (3.70%)	2 (18.18%)	1 (6.25%)
B) From 3 to 6 salaries	2 (7.40%)	4 (36.36%)	8 (50%)
C) From 1 to 3 salaries	23 (85.18%)	4 (36.36%)	7 (43.75%)
D) Up to 1 salary	1 (3.70%)	1 (9.09%)	0 (0.00%)

*Data are expressed as mean and standard deviation for age and relative frequency for the other variables; GNC, Group nutrition counseling; INP, Individualized Nutrition Prescription; ABEP, Brazilian Association of Research Companies*.

All the variables were tested between the groups and neither significant difference was observed before 12-week intervention (*p* > 0.05).

The participants age was similar in the GNC and INP groups (*p* > 0.05).

### Anthropometric and Body Composition Responses

**Figure 2:**
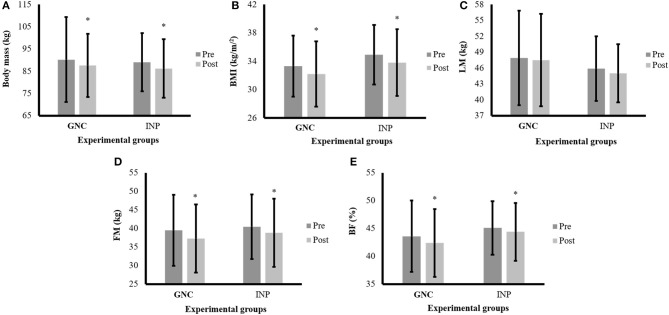
Anthropometric and body composition variables of the participants before and after the intervention period. Note: data are expressed by mean and ± standard deviation; GNC, group nutrition counseling; INP, individualized nutrition prescription; pre, pre-intervention; post, post-intervention; **(A)** body mass responses among two experimental groups; **(B)** = body mass index (BMI) among two experimental groups; **(C)** = learn mass (LM) among two experimental groups; panel **(D)** fat mass (FM) among two experimental groups; **(E)** = body fat percentage (BF) among two experimental groups; *time effect with *p* < 0.05.

Figure 2 presents the anthropometric and body composition variables of the participants.

A time effect was identified for body mass (*F* = 27.05; *p* < 0.001), BMI (*F* = 30.69; *p* < 0.001), FM (*F* = 20.40; *p* < 0.001) and BF (*F* = 6.84; *p* = 0.01), with the *post-hoc* showing lower values after the intervention period (*p* = 0.001) for all comparisons. However, no significant differences were detected for LM (Figure 2). The Cohen's *d* for the different comparisons between the groups was as follows: for BM in GNC, *d* = −0.13 (*small effect*), and INP, *d* = −0.21 (*medium effect*); for BMI, in GNC, *d* = −0.24 (*medium effect*), and INP, *d* = −0.23 (*medium effect*); for LM in GNC, *d* = −0.08 (*small effect*), and INP, *d* = −0.15 (*small effect*); for FM, in GNC, *d* = −0.22 (*medium effect*), and INP, *d* = −0.20 (*medium effect*); and for BF, in GNC, *d* = −0.18 (*small effect*), and INP, *d* = −0.14 (*small effect*).

### Metabolic Responses

Table 4 presents the metabolic variables of women that participated in the present study.

**Table 4:** Metabolic variables of women that participating in the present study.

**Variables**	**GNC (*n* = 16)**			**INP (*n* = 11)**		
	**Pre**	**Post**	***Cohen's d***	**Pre**	**Post**	***Cohen's d***
Insulin (μ/mL)	12.9 ± 13.8	12.8 ± 9.9	0.00	16.1 ± 8.2	14.8 ± 8.7	−0.16
HbA1c (%)	5.4 ± 0.6	5.4 ± 0.4	0.01	5.7 ± 0.9	5.6 ± 0.7	−0.16
Triglycerides (mg/dL)	116.1 ± 61.1	110.4 ± 37.5	−0.09	119.9 ± 47.2	118.0 ± 40.1	−0.04
Total cholesterol (mg/dL)	197.3 ± 43.2	183.3 ± 28.8	−0.32	219.4 ± 22.5	209.8 ± 37.6	−0.42
LDL-c (mg/dL)	113.2 ± 38.9	103.5 ± 26.67	−0.24	138.2 ± 21.9	131.3 ± 26.7	−0.31
HDL-c (mg/dL)	58.3 ± 18.7	56.0 ± 14.9	−0.12	55.0 ± 11.9	50.5 ± 10.5	−0.37
AST (U/L)	23.9 ± 8.8	25.3 ± 12.8	0.15	24.7 ± 5.6	23.5 ± 4.8	−0.21
ALT (U/L)	24.2 ± 8.8	23.0 ± 10.1	−0.12	31.8 ± 11.9	29.1 ± 8.4	−0.22

*Data are expressed by mean and ± standard deviation; GNC, group nutrition counseling; INP, individualized nutrition prescription; pre, pre-intervention; post, post-intervention; HbA1c, glycated hemoglobin; LDL-c, low density lipoproteins; HDL-c, high density lipoproteins; AST, aspartate aminotransferase; ALT, alanine aminotransferase; for the classification of metabolic variables and effect size, please access the methods*.

For insulin, A1C, triglycerides, total cholesterol, LDL-c, HDL-c, AST, and ALT, no significant differences were observed (*p* > 0.05). It is noteworthy that the values for insulin, on average, were within the normal range, and the same was found for HDL-c. The mean A1C level was compatible with the diagnosis of prediabetes in the GNC at the time of the pre-intervention, returning to normal values after the intervention period. Both intervention groups had the mean triglyceride levels within the normal range. However, in the GNC group, mean values of total cholesterol were identified in the borderline classification, while the mean values of the INP group were within the normal range. For LDL-c, the average values of GNC were found to be slightly above the proposed recommendations. Although no significant reductions in total cholesterol and LDL-c were identified, Cohen's d found a moderate effect size for both lipid-profile variables, with lower values after the intervention period. Such findings may be considered positive since there was a reduction in the cardiometabolic risk at post-intervention, in both experimental groups.

### Food Record Data

Table 5 presents the participants nutrition intake before and after 12-week intervention period.

**Table 5:** Food record information of women that participating in this study.

**Diet components**	**GNC (*n* = 16)**			**INP (*n* = 11)**		
	**Pre**	**Post**	***Cohen's d***	**Pre**	**Post**	***Cohen's d***
TCI (kcal/day)*	1637.0 ± 396.6	1272.5 ± 416.9	−0.91	1816.4 ± 747.2	1194.0 ± 367.5	−0.83
TF (g/day)	11.8 ± 5.3	10.9 ± 4.5	−0.18	11.5 ± 5.8	8.9 ± 3.6	−0.46
CHO g/day*	201.7 ± 42.5	149.9 ± 50.9	−1.21	198.1 ± 61.1	154.3 ± 60.8	−0.71
CHO %	50.2 ± 4.7	48.2 ± 7.1	−0.44	47.3 ± 9.1	53.2 ± 8.5	0.64
PTN g/day*	69.4 ± 20.0	62.4 ± 20.1	−0.35	85.7 ± 49.9	53.1 ± 15.0□	−0.64
PTN %	17.5 ± 3.3	19.6 ± 2.7	0.62	18.8 ± 4.2	17.9 ± 2.8	−0.21
PTN g/kg/day	1.4 ± 0.3	1.6 ± 0.5	−0.36	1.4 ± 0.6	1.8 ± 0.6	0.65
LIP g/day*	61.4 ± 22.4	47.1 ± 20.1	−0.64	73.6 ± 42.2	39.6 ± 13.2	−0.80
LIP %	32.3 ± 6.4	32.6 ± 7.5	0.04	34.3 ± 6.3	29.3 ± 7.0	−0.79
S. F. (g/day)*	17.1 ± 9.1	13.5 ± 6.6	−0.40	24.4 ± 17.8	11.7 ± 6.2?	−0.71
P.F (g/day)*	10.4 ± 4.9	8.4 ± 4.4	−0.41	10.2 ± 4.5	5.8 ± 2.2	−0.98
M. F. (g/day)	26.7 ± 9.7	13.7 ± 6.8	−1.35	22.8 ± 17.8	11.8 ± 4.9	−0.61

GNC, group nutrition counseling; INP, individualized nutrition prescription; pre, pre-intervention; post, post-intervention; TF, total fiber; F, fat; S, saturated; P, polyunsaturated; M, monounsaturated; TCI, total caloric intake; CHO, carbohydrate; PTN, protein; LIP, lipid; ^*^time effect, with pre-intervention different from post-intervention (p < 0.05); □ = interaction, with lower values for the NOG when compared to itself after the intervention (p = 0.01); for the classification of the effect size, please access the methods.

A time effect was identified for TCI (*F* = 43.30; *p* < 0.001), CHOs in grams (*F* = 24.11; *p* < 0.001) and LIPs in grams (*F* = 21.12; *p* < 0.001), with the *post-hoc* showing lower values after the intervention period (*p* = 0.001), for all comparisons. For polyunsaturated fat, a time effect was observed (*F* = 8.24; *p* = 0.008), with the *post-hoc* showing lower values after the intervention period (*p* = 0.01). For PTNs, a time effect (*F* = 10.35; *p* = 0.003) was identified with the *post-hoc* showing lower values after the intervention period (*p* = 0.007). In addition, an interaction was detected (*F* = 4.33; *p* = 0.047), in which the *post-hoc* showed lower values in the GNC than in the INP after the intervention period (*p* = 0.01). For saturated fat, a time effect (*F* = 15.80; *p* < 0.001) was verified, with the *post-hoc* indicating lower values after the intervention period (*p* = 0.001). Additionally, an interaction (*F* = 4.79; *p* = 0.038) was identified with the *post-hoc*, indicating lower values in the GNC group at the post-intervention than values in that group at the pre-intervention period (*p* = 0.002). However, no significant differences were observed for %LIPs, %CHOs, %PTNs, PTNs g/kg/day, fiber consumption, and monounsaturated fat intake (*p* > 0.05).

### Questionnaires Responses

**Figure 3:**
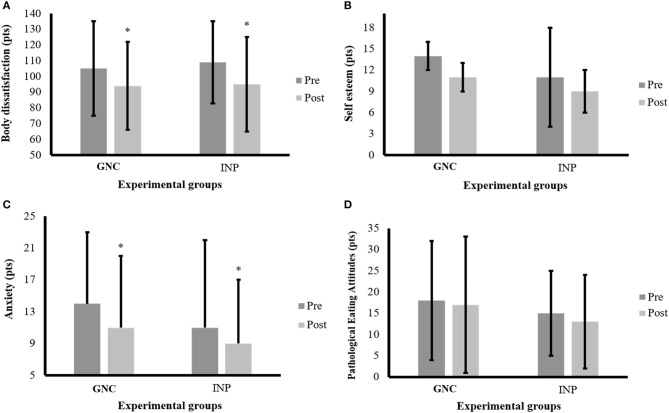
Mental health variables of the participants before and after the intervention period. Note: data are expressed by mean and ± standard deviation; GNC = group nutrition counseling; INP, individualized nutrition prescription; pre, pre-intervention; post, post-intervention; **(A)** body dissatisfaction responses; **(B)** Self-esteem responses; **(C)** anxiety responses; **(D)** pathological eating attitudes responses; *time effect with *p* < 0.05.

The Figure 3 presents the results of mental health tests for the women participating in this study.

For the IPAQ, no significant differences were observed after the intervention period (*p* > 0.05). The only differences observed were on the days of the programmed physical exercises, i.e., on Mondays, Wednesdays, and Fridays. For body image dissatisfaction, only a time effect was identified (*F* = 7.1; *p* = 0.013), with the *post-hoc* showing lower values after the intervention period (*p* = 0.011), and the effect size for the GNC was *d* = −0.53 (*medium effect*), whereas for the INP, an effect size of *d* = −0.36 (*medium effect*) was found. For anxiety, only a time effect was detected (*F* = 8.2; *p* = 0.008), with the *post-hoc* indicating lower values after the intervention period (*p* = 0.004), with effect sizes of *d* = −0.54 (*medium effect*) and *d* = −0.33 (*medium effect*) in the GNC and INP, respectively. However, no significant differences were identified in self-esteem (*p* > 0.05), which presented an effect size of *d* = 0.14 (*small effect*) in the GNC and *d* = −0.50 (*medium effect*) in the INP. Finally, there were no significant differences regarding pathological eating attitudes (*p* > 0.05), which presented an effect size of *d* = −0.20 (*small effect*) for the GNC and *d* = −0.07 (*small effect*) for the INP.

## Discussion

Considering that the objective of the present study was to investigate the effects of two different nutritional strategies, namely, individualized nutritional prescription with calorie restriction and group nutritional orientation, combined with the practice of concurrent exercises on anthropometric parameters, body composition, metabolic variables, food intake and mental health of overweight and/or obese women, the main results indicated: (1) reductions in body mass, BMI and absolute and relative fat mass in both groups; (2) absence of differences in LM in both groups; (3) absence of differences in metabolic parameters in both groups; (4) reductions in total caloric intake/TCI (kcal/day), LIP, saturated and polyunsaturated fat in both groups; (5) adequacy of CHO and PTN macronutrients in the pre- and post-intervention periods; (6) reductions in body dissatisfaction and anxiety in both groups; and (7) no significant differences in self-esteem nor in pathological eating attitudes in both groups.

Reducing body mass, BMI and body fat in overweight and obese people is essential to promote health improvement, thus decreasing the prevalence of chronic diseases in a population context (18, 19). According to Swift et al. (46), the inclusion of a physical training program in the treatment of obesity is fundamentally relevant to maintain long-term body mass control. Similar findings were also reported by Madjd et al. (15), who demonstrated that regular exercise increased caloric expenditure, reduced body adiposity, and improved lipid profile and insulin resistance. Although modest reductions in body mass and BMI (~3 kg and 1 kg/m^2^, respectively) were observed in the present study, the loss of body fat (~2 kg) was associated with the maintenance of LM, which is a positive result based on the age of the women investigated. Physiologically, there is a loss of muscle mass with the aging process, which can directly affect functional capacity and contribute to a reduction in RMR (47). Reductions in LM can be minimized through physical exercise and nutritional monitoring (47). However, muscle mass gain with physical training may be different in everyone based on the type of training, somatotype and motivation for physical activity.

Regarding metabolic parameters, in general, no changes were observed after the 12-week intervention period. Considering the average laboratory results for all participants, it can be concluded that the studied population presents obesity with a healthy metabolic profile, except for some women in the GNC group (48). Mean total cholesterol and LDL-c levels were found in the borderline classification in the GNC group. Previous evidence has indicated that the prevalence of obesity with a metabolically healthy profile was between 2 and 28% of the population (49). This condition is more prevalent in women, due to the predominance of a gynecoid pattern distribution in this gender, and less prevalent in the elderly, due to the redistribution of subcutaneous to visceral fat that occurs in the aging process (49). However, medicine considers the so called “metabolically healthy obesity” with some parsimony as overweight may progress with the development of insulin resistance, changes in lipid profile, osteoarticular diseases and cancer, among other complications (50).

An interesting finding of this study was that the mean values of A1C in the GNC group, compatible with the diagnosis of prediabetes in the pre-intervention period, returned to normal values after the intervention; a situation previously demonstrated in diabetes prevention studies. Robust scientific evidence has indicated that lifestyle changes are highly effective in preventing the progression of prediabetes to diabetes and are more powerful than drugs such as metformin (51). Therefore, behavioral changes and the adoption of a healthy and active lifestyle are essential strategies for health promotion and the fight against the current diabetes epidemic that is closely associated with the high prevalence of obesity.

One of the limitations of this study was the possibility of an underestimation of the energy intake from the dietary records. The method chosen to quantify food intake and obtain valid and reliable data from real-world nutritional studies is a difficult task as there is no gold standard for this assessment, and all existing methods are subject to variation and measurement errors (52). This underestimation may partly explain the low-calorie values reported in the dietary records of the two groups in the present study. Nutrition guidelines state that for healthy individuals, a balanced diet should consist of 45 to 65% CHO, 15 to 25% PTN, and up to 30% LIP of the TCI (34). Therefore, the adequacy of protein intake may be explained by the increased consumption of meat, milk and dairy products among Brazilians in the last three decades (53, 54). The percentage of CHO can also be explained by the trend observed in the results of the Family Budget Survey (53). This survey showed an increased in the intake of foods rich in fat, sugar, with low amounts of fiber by the Brazilian population, especially among those with lower purchasing power. On the other hand, according to same survey (53), the consumption of complex CHO, such as rice, beans and pulses was decreased in the Brazilian diet. This fact may explain the low fiber intake found in the present study, as the nutritional calculation program used to quantify food intake does not distinguish between simple and complex CHO. The reduction in the consumption of these macronutrients can explain the decrease in TCI consumption reported by the participants.

Different dietary patterns modulate multiple aspects of the atherosclerotic process and cardiovascular risk factors. For example, saturated and trans-fat intake are associated with increased plasma LDL-c, and the replacement of saturated dietary fat for monounsaturated or polyunsaturated fat may improve the lipid profile, lowering cardiovascular risk (54). In the present study, both groups had a high intake of saturated fat, which was significantly reduced after the intervention, regardless of the nutritional method. It was also observed that there was an inadequate intake of monounsaturated and polyunsaturated fatty acids, which reflected the poor food quality consumed by the participants, which in the long run may contribute to the onset of CVD (55). The low consumption of monounsaturated and polyunsaturated fatty acids in the study may be related to the low purchasing power of the participants. In Brazil, foods rich in fatty acids, such as olive oil, canola oil, fatty fish and nuts, are quite expensive.

People who are overweight and/or obese are more likely to develop psychological problems, including body image dissatisfaction. Body image goes hand in hand with mental health, and with improvements in this parameter, the individual becomes more prone to maintain treatment (56). Thus, reducing body dissatisfaction is certainly a motivational factor in the process of behavior change. The determinants of a positive or negative body image depend on factors such as age, gender, media exposure, cultural, social values and beliefs. Different stages of life imply different yearnings for the desired body pattern (57). Thus, in the case of obese middle-aged women, exposed to the standards of beauty imposed by the media, improvements in their body acceptance was extremely relevant after the intervention period.

The relationship between anxiety and obesity is bidirectional with one condition affecting the other. For instance, individuals with genetics favorable to weight gain eat more when exposed to stress and anxiety (58). The aesthetic imposition of body worship also generates anxiety and frustration in obese people. There is the following premise imposed by society “the obese could only be happy being thin,” and instead of weight loss, these types of thoughts can worsen self-esteem and cause self-image dissatisfaction (59, 60). The findings of the present study corroborate the study by Bansal et al. (61), which states that people with obesity present major psychological problems, and that, it seems to be a relationship between anxiety, pathological eating attitudes and obesity.

In the behavioral field, anxious people tend to move away from social life, and exercise could represent a way of engaging these individuals in group activities (62). A systematic review had identified that physical activity improved symptoms related to depression, anxiety, postpartum depression, among other aspects (63). In the present study, no significant differences were observed for self-esteem and pathological eating attitudes. These findings may be explained by the short intervention period and the complexity of factors that influence the self-esteem and the possibility of change (64, 65). Because those in the present study were middle-aged and predominantly low-income women, they may have come under less pressure from society and the media to have slim, perfect bodies, unlike younger, higher-income women. Moreover, this sample did not present, on average, significant eating disorders. It should also be considered that the instrument used may not be sensitive to specifically tracking the unique characteristics of the overweight or obese population.

It was evident from a previous study that women with little education or less favorable economic class were more likely to develop obesity and psychological disorders (7). The differences in self-perception that women belonging to each social class have about their own bodies is one of the factors that interferes with the choice of food and its relationship with health and beauty. da Santos (6) considered that low-income groups prioritize the ability to work and body strength over the body shape. Thus, low-income women are more susceptible to overweight, low self-esteem and body image distortion and often find themselves in a frustrating situation with the financial inability to acquire the means to lose weight and engage in physical exercise, such as access to gym, personalized diets and aesthetic treatments. Faced with the belief that the picture is irreversible, they focus their anxieties on the pleasures of food, giving immediate expression to their frustrations (7).

Given the high prevalence of obesity and psychological disorders in underprivileged social classes, as reported above, the short intervention time and the considerable gains in reducing anxiety and decreasing body image dissatisfaction demonstrate that the present study may be a cost-effective measure for use in public health. The findings are also consistent with previous evidence that small changes in food intake and lifestyle are critical to promote moderate and sustainable medium-term weight loss (66). Thus, multiprofessional programs focusing on weight loss that include physical activity and nutritional counseling can lead to reductions in direct and indirect costs associated with obesity treatment, as a larger number of people can be served at a relatively lower cost, especially in emerging countries such as Brazil, which has been constantly suffering from the scarcity of resources to treat chronic diseases, especially obesity.

Other authors have proposed strategies focused on reducing body mass, specifically in women. Monteiro et al. (67) identified that nutrition education performed in conjunction with regular and structured exercises were more likely to present satisfactory results when compared to nutrition education programs alone. In addition, data from a meta-analysis published in 2018 showed significant reductions in body mass and body fat in premenopausal and postmenopausal women after nutritional and physical interventions (68). Therefore, for the success of a weight loss program, it is necessary to prescribe food or nutritional guidance (10–13), as well as the incorporation of physical exercises, especially strength exercises, to maintain or increase muscle mass.

Therefore, GNC and INP interventions, as well as competing exercises with similar groups, may be relevant strategies to promote body self-acceptance and anxiety reduction in women. Thus, group nutritional orientation activities combined with concurrent exercise with low-cost equipment can be incorporated by public and private health systems to promote health and reduce the impacts of obesity on middle-aged women. As the two nutritional approaches studied showed similar results, it is suggested that resistance exercise associated GNC may be an economic strategy to combat obesity at the population level, as multiple people can be treated together, minimizing public health costs. In addition, the configuration of these programs is feasible given the low-cost of the equipment needed to perform resistance exercises.

Additionally, the applied method can be tested in other settings, such as primary health care facilities, community centers, and hospitals, as responses were similar in both intervention groups. In view of the points listed, the choice of the nutritional method should be based on the experience and capacity of health professionals as well as the program participant's preference. Besides that, this study could be replicated in larger samples in order to confirm these results. The results cannot be generalized to other populations because of the small sample size. However, this research could be replicated in larger samples to confirm these results particularly in developing countries where the wages are relatively low. It may be cost-effective to hire exercise trainers and nutritionists to deliver a similar intervention on a larger scale if health complications could be prevented. As a possible limitation, we can mention the lack of a control group, although Hecksteden et al. (69) considered that it would not be ethical to use a randomized control group in this situation. We must consider that we are trying to treat a disease that is considered the pandemic of the twenty first century. Therefore, both groups need to receive the benefits of the intervention. As a final consideration, a 12-week intervention is the first point to promote changes in a weight loss program (67, 68) following robust guidelines. Because of this, new studies with several months and even, years, could be investigated to analyze these responses in the long-term.

## Conclusion

Finally, it is concluded that nutritional counseling or prescription, associated with resistance exercise, can be effective in reducing body mass, BMI, FM, BF, anxiety and body dissatisfaction, as well as the maintenance of LM. Thus, the determination of nutritional intervention should be based on the individual's profile and respect for their wishes, which increases the likelihood of adherence to the chosen nutritional program and the maintenance of long-term dietary changes.

## Practical Applications

The proposed lifestyle intervention program presents aspects of originality regarding nutritional strategies aimed at weight loss. Nutritional counseling based on food choices has been proven to be as effective as delivering a quantity-based eating plan. Interdisciplinarity was practiced, and the psychoeducation techniques used by both nutrition professionals and physical education professionals promoted changes in behavior that may result in improved quality of life. Since the availability of health professionals is limited and poorly distributed in Brazil and other emerging countries with similar public health systems, it seems reasonable to assume that the constitution of groups of patients with the same obesity profile is an alternative to promote an improvement in quality of life. In addition to representing a low-cost strategy, the exposure of an individual to multiple perceptions generates discussion and reflection and points out ways to make patients feel safer and supported. In contrast, for the success of any weight loss program, the pleasure of performing the proposed activities must be substantial. In this sense, topics such as barriers to physical activity, how to adopt a more active lifestyle, and working with experiences and pleasant memories of physical activity should be more intensely debated. As a suggestion, the present study proposes expanding to other age groups and sexes and evaluating the impact of family influences on eating habits and physical inactivity.

## Data Availability Statement

The datasets generated for this study are available on request to the corresponding author.

## Ethics Statement

The study was approved by the Ethics and Research Committee of the State University of Maringa by Opinion No. 2,655,268/2018. This study was also submitted and approved by Brazilian Trials Clinical Studies (REBEC) by the Health Minister under register RBR2yzs76. The patients/participants provided their written informed consent to participate in this study.

## Author Contributions

BB drafted the initial manuscript and conducted the data analysis. MB assisted in the drafting of the manuscript. CF, AF, and RB led the data collection team and critically revised the manuscript for intellectual content. BB, MB, CF, AF, RB, SL, NJ, and SB critically revised the manuscript for intellectual content. BB, MB, and NJ designed the study, conducted some analyses, and critically revised the manuscript for intellectual content. All authors contributed significantly to the interpretation of the data.

## Conflict of Interest

The authors declare that the research was conducted in the absence of any commercial or financial relationships that could be construed as a potential conflict of interest.
